# Ultrasound (US)-Guided Perineural Amniotic Membrane and Umbilical Cord Particulate Injections for Iatrogenic Radial Nerve Palsy

**DOI:** 10.7759/cureus.70949

**Published:** 2024-10-06

**Authors:** Mohammed Gartit, Abdelilah Rhoul, Mohamed Noumairi, Ahmed Amine EL Oumri

**Affiliations:** 1 Physical Medicine and Rehabilitation, Mohammed VI University Hospital, Oujda, MAR; 2 Faculty of Medicine and Pharmacy, Mohammed Ist University, Oujda, MAR; 3 Faculty of Medicine, Mohammed Ist University, Oujda, MAR

**Keywords:** am/uc particulate, iatrogenic radial nerve palsy, regenerative medicine, rehabilitation, us-guided injection

## Abstract

Radial nerve injury is a common occurrence in the upper extremities, with various treatment options available such as neurolysis, nerve grafts, or tendon transfers. Recently, amniotic membrane and umbilical cord (AM/UC) particulates have emerged as promising treatments for this type of nerve pathology. Here, we report a new case involving a 24-year-old man who experienced total paralysis of the radial nerve following a humerus shaft fracture. He was treated with peri-nerve injections of AM/UC and underwent an intensive physical rehabilitation program. Three months after the initial injury, significant progress was observed in both motor and sensory functions of his radial nerve

## Introduction

Radial nerve palsy is a common complication of orthopedic procedures involving the upper extremity, such as humeral shaft surgery. The classification of radial nerve palsy in conjunction with humerus fracture depends on whether the nerve damage occurred at the time of the fracture (primary) or during treatment (secondary). A meta-analysis of 4,517 fractures found that the incidence of primary radial nerve palsy was 11.8% [1].

In this case report, we present a patient who developed iatrogenic radial nerve palsy (IRNP) following a humerus shaft fracture. The patient experienced severe motor weakness and loss of sensation in the radial nerve territory of the affected arm. Not all nerve injuries require surgical repair, especially in cases of prolonged traction lesions [2-3].

The patient was treated with peri-nerve injections of AM/UC particulate and underwent an intensive rehabilitation program, resulting in significant improvement in motor and sensory functions three months after initial admission.

## Case presentation

**Figure 1:**
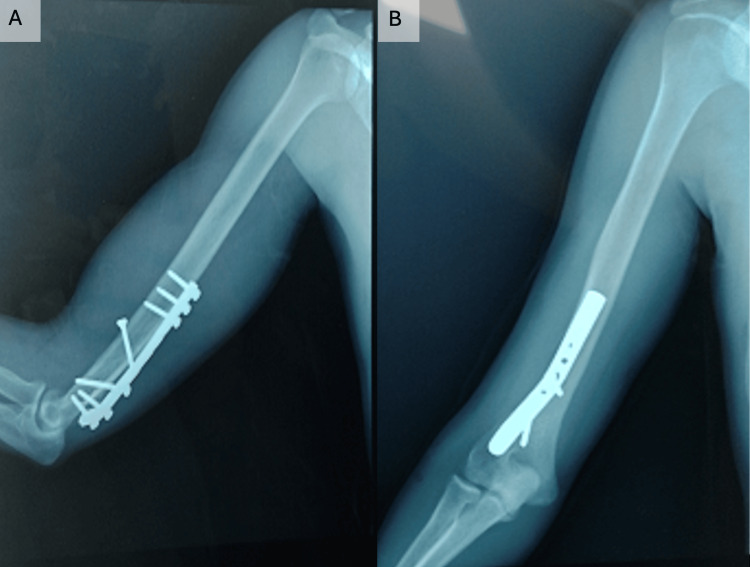
One month after fixing the fracture of the distal end of the humeral shaft with a screw plate, an X-ray was taken to monitor/control the healing process. (A) Profile impact, (B) front impact

A 24-year-old man with a history of humeral shaft fracture of the left arm sustained during an arm-wrestling match presented with no sensory or motor deficit in the radial nerve territory before undergoing surgery two days post-fracture. The surgical procedure involved osteosynthesis using a screw plate and a posterior tricipital approach. Following surgery, the patient was referred to our department for rehabilitation (Figure 1).

Upon physical examination, the patient exhibited total functional impairment of the wrist and hand, with an inability to extend the wrist and fingers. Muscle strength assessment using the Oxford Scale (OS) showed a level of 0/5 for the wrist and finger extensor muscles. Additionally, he reported neuropathic pain in the radial nerve territory, although sensory examination revealed complete anesthesia below the lower arm extremity in this territory.

**Figure 2:**
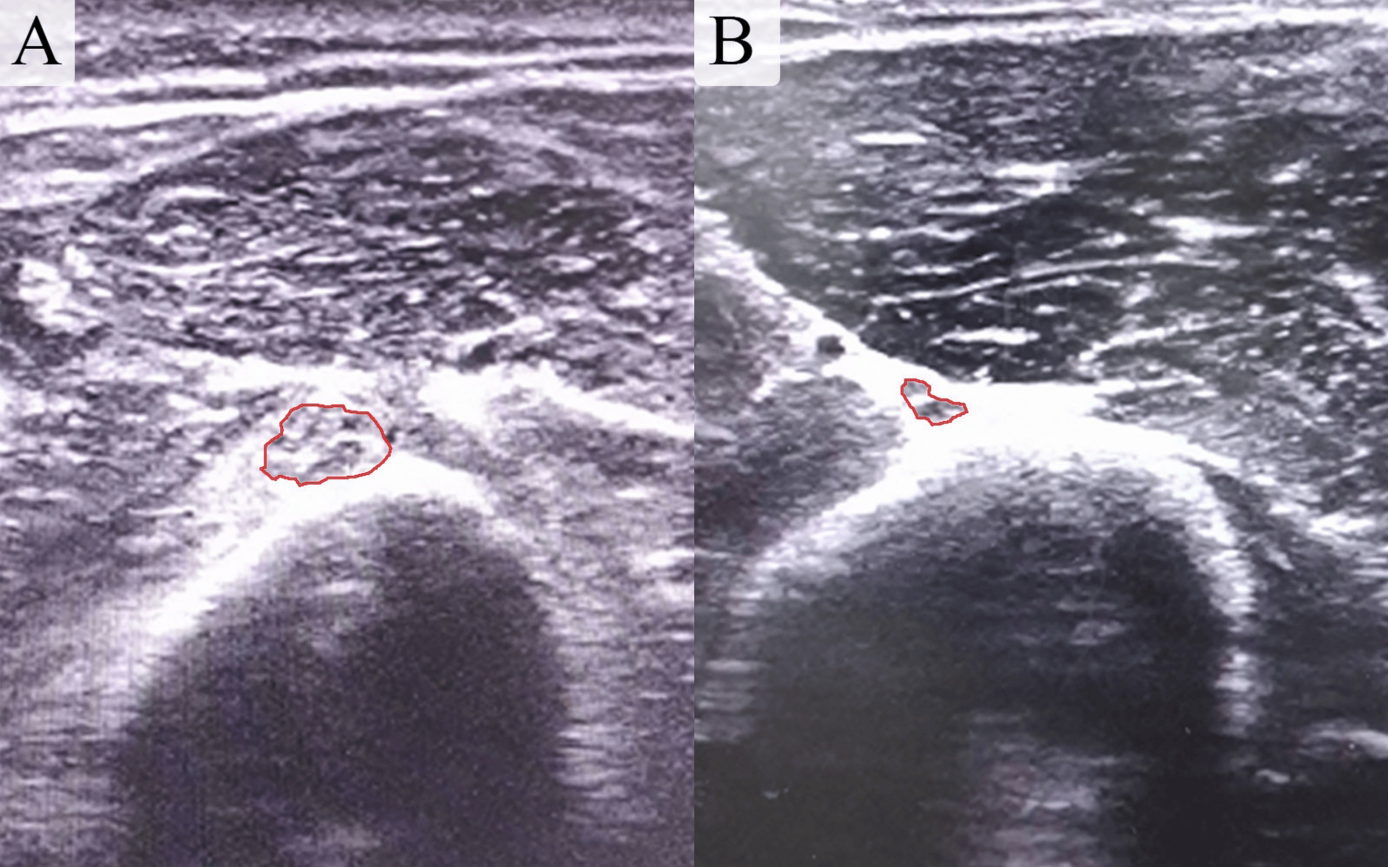
Enlarged images obtained through comparative ultrasound scans of the left and right arms reveal a damaged radial nerve with a cross-sectional area (CSA) of 0.22 cm² in the left arm (image A), in contrast to a normal radial nerve with a CSA of 0.05 cm² in the right arm (image B).

An electromyography study of the radial nerve indicated truncal damage, while an ultrasound (US) examination revealed an intact but enlarged nerve lacking its distinct fascicular appearance, suggestive of iatrogenic radial nerve palsy due to prolonged traction (Figure 2).

Despite initiating an intensive rehabilitation program, the patient did not show significant improvement. Subsequently, a peri-nerve injection of AM/UC particulate was performed under US guidance two months post-surgery to facilitate nerve regeneration and improve muscle strength and function.

Ultrasound-guided injection technique

**Figure 3:**
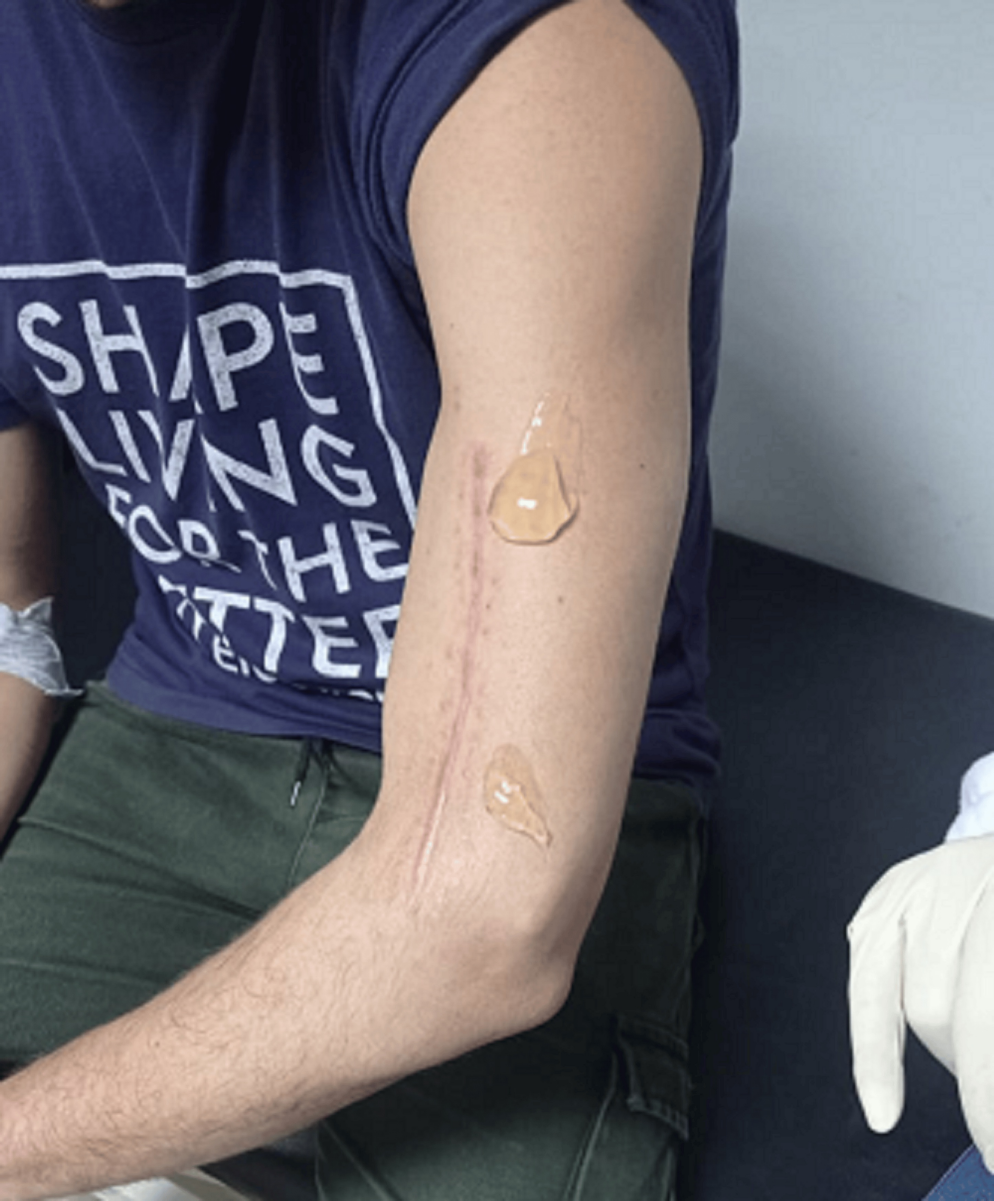
Preparation of the first US-guided perineural injection of AM/UC particulate for damaged radial nerve AM/UC: Amniotic membrane/umbilical cord

A vial containing 25 mg of AM/UC particulate was diluted with 10 cc of 0.9% isotonic saline, resulting in a total volume of 10 cc. The mixture was drawn into a syringe equipped with a 5 cm 18G needle. The radial nerve was visualized using ultrasound, employing a high-frequency linear probe with a frequency range of 8-12 MHz. The injectate was administered around the enlarged segment of the nerve using an in-plane syringe visualization technique (Figure 3).

Clinical evolution after the first injection

One month after the injection, a significant reduction in neuropathic pain was observed. Additionally, there was an improvement of 2 points in wrist extensor muscle strength and 1 point in finger extensor muscle strength according to the OS. Sensory examination also showed improvement.

Another month later, the motor score for the wrist extensor muscles improved to 3 out of 5, while it was 1 out of 5 for the finger and thumb extensor muscles. The patient did not report any neuropathic pain.

**Figure 4:**
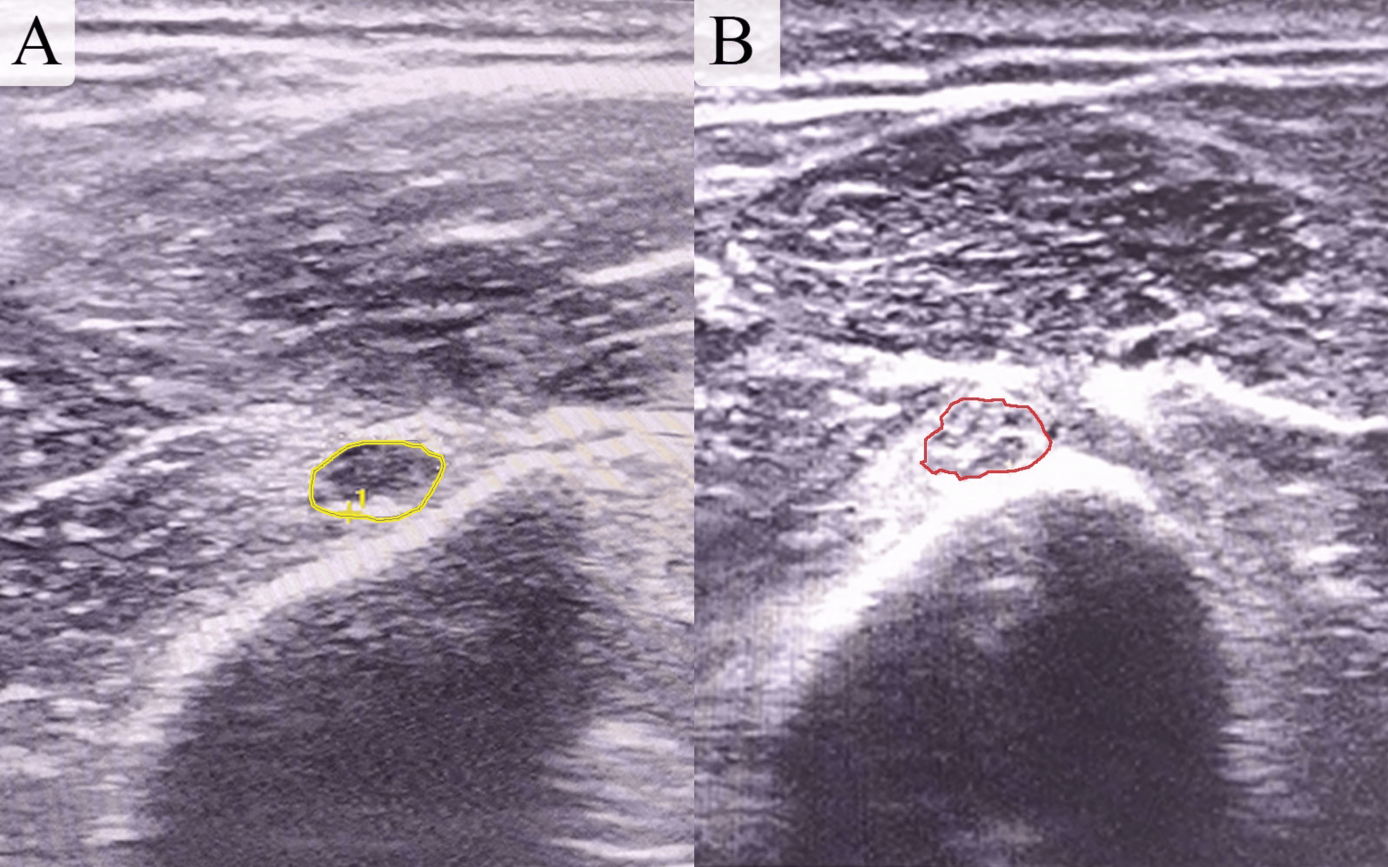
Comparative ultrasound images show a decrease in swelling and a reduction in the cross-sectional area (CSA) of the radial nerve, which decreased from 0.22 cm² at the time of the first injection (image B) to 0.13 cm² two months later (image A).

Ultrasound evaluation of the radial nerve indicated an improvement in its condition, with a decrease in swelling and a reduction in the diameter of the enlarged portion from 0.22 to 0.13 cm² in cross-sectional area (CSA) (Figure 4).

**Figure 5:**
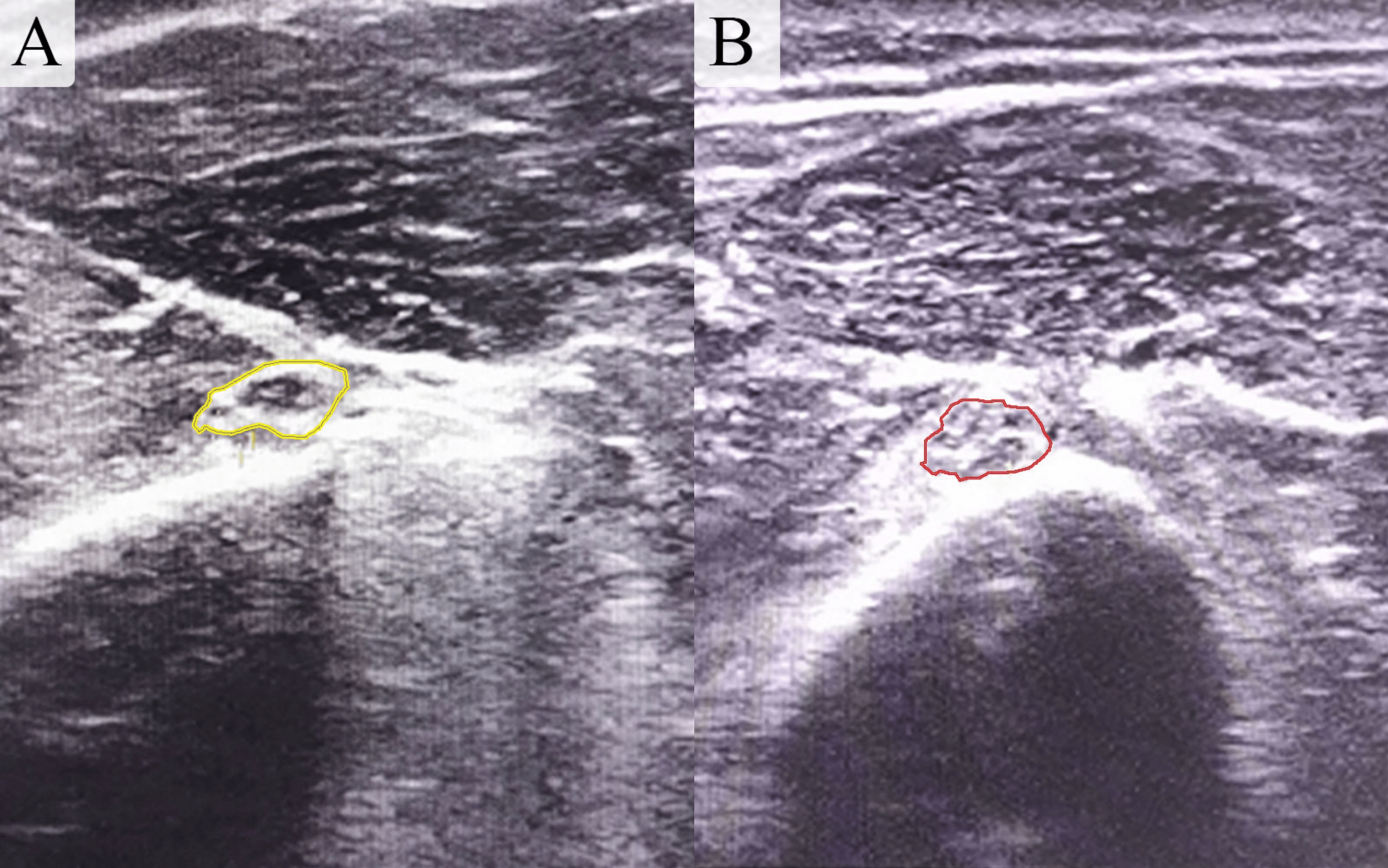
Comparative ultrasound images show a reduction in swelling and cross-sectional area (CSA) of the nerve, which decreased from 0.22 cm² at the time of the first injection (image B) to 0.11 cm² three months later (image A).

By the third month, the patient continued to improve in motor function, with extensor muscle strength rated 4/5 on the OS scale and 2/5 for the finger and thumb extensor muscles. Ultrasound evaluation showed a further decrease in the swollen portion of the nerve, which reduced from 0.13 to 0.11 cm² (Figure 5).

## Discussion

Several cases of iatrogenic radial nerve palsy (IRNP) have been reported in the literature [4-6]. In this case, the radial nerve was likely traumatized during the surgical approach and placement of the osteosynthesis plate. Positive results with AM/UC have been reported in ophthalmologic use for patients with neuropathic corneal pain [7-9]. In orthopedics, AM/UC has been utilized as a conduit or wrap to promote neural regeneration, improve functional outcomes, nerve histomorphometry, and reduce pain [10-12]. Micronized AM/UC has also been injected in-office to deliver similar therapeutic benefits due to its rich concentration of growth factors, cytokines, and other extracellular matrix components, including neurotransmitters and neurotrophic factors [13,14].

The clinical recovery time after IRNP varies, with a median time of 16 weeks and a range of 5 to 30 weeks [15]. In our case, clinical improvement, particularly in the wrist extensor muscles, was observed four weeks after administering AM/UC via perineural injection. This suggests that perineural injection of AM/UC could potentially promote radial nerve regeneration and reduce the recovery time of iatrogenic radial nerve palsy.

Furthermore, Li et al. conducted a study to evaluate the effectiveness of human amniotic membrane transplantation with human umbilical cord mesenchymal stem cells for treating radial nerve injury [16]. After 12 weeks, over 80% of patients who received the cell transplantation showed significant improvements in muscular strength, touch, and pain sensations, compared to only 55-65% of patients in the control group. Additionally, the transplantation group demonstrated significantly better muscular electrophysiological function in the region dominated by the injured radial nerve at 8 and 12 weeks, as compared to the control group. It's important to note that this was a non-randomized, concurrent controlled study.

In a retrospective study assessing the safety and effectiveness of AM/UC particulate in treating neuropathic pain in the lower extremities, 16 patients who received perineural injections of the particulate were involved. The results indicated that after an average of 2.7 injections per extremity, symptoms improved by 30.0 ± 24.5% at 1 week, 46.6 ± 29.9% at 1 month (P < .005), 70.7 ± 14.3% at 2 months (P < .001), 72.3 ± 16.9% at 3 months (P < .001), and 61.0 ± 34.4% at 5-6 months (P < .01). No adverse events or complications were observed regarding the injection of AM/UC particulate [17]. In our case, the neuropathic pain related to IRNP was completely resolved four weeks after perineural injection of AM/UC particulate, with no reported complications or adverse events.

## Conclusions

While iatrogenic radial nerve palsy (IRNP) is a recognized complication of humerus fracture surgery, this case report underscores the significance of utilizing ultrasound exploration to potentially yield valuable insights that can substantially influence patient treatment and prognosis. Additionally, the favorable clinical outcome observed in this case, coupled with findings from prior studies, lends support to the utilization of amniotic membrane and umbilical cord (AM/UC) as a safe and versatile biological product. Nonetheless, further research is warranted to comprehensively assess the efficacy of AM/UC particulate in managing this condition.
